# Role of Pannexin-1-P2X7R signaling on cell death and pro-inflammatory mediator expression induced by *Clostridioides difficile* toxins in enteric glia

**DOI:** 10.3389/fimmu.2022.956340

**Published:** 2022-08-22

**Authors:** Andrea V. Loureiro, Lauro I. Moura-Neto, Conceição S. Martins, Pedro I. M. Silva, Matheus B.S. Lopes, Renata F. C. Leitão, Juliana M. Coelho-Aguiar, Vivaldo Moura-Neto, Cirle A. Warren, Deiziane V.S. Costa, Gerly A. C. Brito

**Affiliations:** ^1^ Department of Morphology, School of Medicine, Federal University of Ceará, Fortaleza, Ceará, Brazil; ^2^ Paulo Niemeyer Brain Institute, Federal University of Rio de Janeiro, UFRJ, Rio de Janeiro, Rio de Janeiro, Brazil; ^3^ Division of Infectious Diseases and International Health, University of Virginia, Charlottesville, VA, United States; ^4^ Department of Physiology and Pharmacology, School of Medicine, Federal University of Ceara, Fortaleza, Ceara, Brazil

**Keywords:** Clostridioides difficile, Clostridioides difficile infection, Pannexin-1, P2X7R, Enteric glia

## Abstract

*Clostridioides difficile* (*C. difficile)* produces toxins A (TcdA) and B (TcdB), both associated with intestinal damage and diarrhea. Pannexin-1 (Panx1) channels allows the passage of messenger molecules, such as adenosine triphosphate (ATP), which in turn activate the P2X7 receptors (P2X7R) that regulate inflammation and cell death in inflammatory bowel diseases. The aim of this study was to verify the effect of *C. difficile* infection (CDI) in the expression of Panx1 and P2X7R in intestinal tissues of mice, as well as their role in cell death and *IL-6* expression induced by TcdA and TcdB in enteric glial cells (EGCs). Male C57BL/6 mice (8 weeks of age) were infected with *C. difficile* VPI10463, and the control group received only vehicle per gavage. After three days post-infection (p.i.), cecum and colon samples were collected to evaluate the expression of Panx1 by immunohistochemistry. *In vitro*, EGCs (PK060399egfr) were challenged with TcdA or TcdB, in the presence or absence of the Panx1 inhibitor (10Panx trifluoroacetate) or P2X7R antagonist (A438079), and Panx1 and P2X7R expression, caspase-3/7 activity and phosphatidylserine binding to annexin-V, as well as *IL-6* expression were assessed. CDI increased the levels of Panx1 in cecum and colon of mice compared to the control group. Panx1 inhibitor decreased caspase-3/7 activity and phosphatidylserine-annexin-V binding, but not *IL-6* gene expression in TcdA and TcdB-challenged EGCs. P2X7 receptor antagonist accentually reduced caspase-3/7 activity, phosphatidylserine-annexin-V binding, and *IL-6* gene expression in TcdA and TcdB-challenged EGCs. In conclusion, Panx1 is increased during CDI and plays an important role in the effects of *C. difficile* toxins in EGCs, participating in cell death induced by both toxins by promoting caspase-3/7 activation *via* P2X7R, which is also involved in IL-6 expression induced by both toxins.

## Introduction

*Clostridioides difficile* (*C. difficile*) is a gram-positive, spore-forming, toxin-producing, anaerobic bacillus and is one of the main causes of antibiotic use-associated nosocomial diarrhea (1, 2) exhibiting an incidence of 8.3 C*. difficile* infection (CDI) cases per 10000 patient-day, costing approximately US $ 4 billion per year in the US (3). The main virulence factors of *C. difficile* are toxin A (TcdA), toxin B (TcdB) and binary toxin (CDT) (4, 5). The intestinal disease caused by CDI can range from mild diarrhea to fulminant disease (2).

The enteric nervous system (ENS) is composed by enteric neurons and enteric glial cells (EGCs) that together control the intestinal reflex (peristalsis), secretion and inflammatory response (6). Enteric glial cells (EGCs), as an important cellular component of ENS, play a key role in regulating intestinal homeostasis, inflammatory response due its ability in interacting with other intestinal cells, as well as in responding to bacteria stimuli (7–11). Complete ablation of EGCs promoted fulminant colitis in mice (12), showing the potential role of these cells in regulating key functions in the gut. It is known that TcdB induces EGCs death *via* reactive oxygen species (ROS)/nicotinamide adenine dinucleotide phosphate (NADPH) oxidase (NOX)/c-Jun N-terminal kinase (JNK) in a caspase-dependent manner but mitochondria-independent pathway, as well as senescence (13–15). *In vitro*, co-culture of ileum mucosal and submucosal layer challenged with TcdB presented higher IL-8 secretion than the culture of each layer alone (16). The fact that EGCs can be found on these two layers (6), suggest a potential role of these cells in the inflammatory response to *C. difficile* toxin.

Pannexin-1 (Panx1) is a well-characterized membrane protein that participates in cell-extracellular environment communication (17–19) and is part of an important mechanism for release of ATP (adenosine triphosphate), an endogenous P2X7 receptor (P2X7R) agonist, to the extracellular environment. P2X7R is involved in many cellular functions, such as metabolism, proliferation, migration, neurotransmitters release and cytokine synthesis (20–24). In the gut, P2X7R can be found in epithelial cells, neurons, macrophages, T cells and EGCs (25–27). Activation of P2X7R begins with the release of ATP through Panx1 channels, which are activated *via* Ca2+ signaling (6, 28). In high levels, ATP *via* P2X7 activation, promotes tissue damage by stimulating cell death (29). In a colitis murine model, activation of P2X7R in EGCs resulted in inflammatory response and cell death (28). However, it is still unknown the role of Panx1 and P2X7R in EGCs death and inflammatory response induced by *C. difficile* toxins.

Given that TcdA and TcdB induces cell death (13, 14) and upregulation of pro-inflammatory mediators (such as IL-6) in EGCs (30), we investigated whether Panx1/P2X7R signaling is involved in these deleterious effects.

## Materials and methods

### Mice

Male C57BL/6 mice (Jackson Laboratory, Farmington, US, 8 weeks old) were housed in temperature-controlled rooms under 12 h light-dark cycles. The animals received water and food ad libitum. All surgical procedures and treatments performed with C57BL/6 mice were conducted in accordance with the Guidelines for Institutional and Animal Care and Use of the University of Virginia, Charlottesville, US. The protocol has been approved by the committee on the Ethics of Animal Experiments of the University of Virginia (Protocol number: 4096).

### *C. difficile* infection model

**Figure 1:**
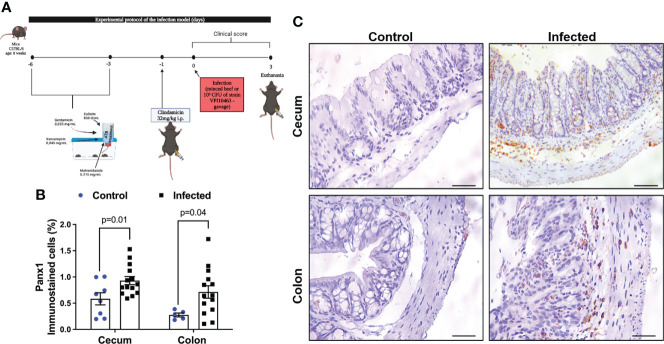
CDI increases Panx1 expression in mouse colon and cecum samples. **(A)** Experimental protocol of the infection model. **(B)** CDI increases Panx1 immunostaining in the cecum and colon of mice. The data are the mean ± SEM of the percentage of the immunopositive area for Panx1 in the cecum and colon of mice submitted to CDI and uninfected in relation to the total area; t test of Student; the value of p is represented in the graph. **(C)** immunohistochemical imaging for Panx1 in mouse colon and cecum tissues with CDI (Bars=100 μm).

Our murine CDI model is broadly used due to the ability to mimic severe diarrhea presented by humans and was performed as previously described (31–35). C57BL/6 mice (n=6 for each group) received an antibiotic treatment (0.035 mg per mL gentamicin, 850 U per mL colistin, 0.215 mg per mL metronidazole, and 0.045 mg per mL vancomycin) in the drinking water for 3 days. After 1 day off antibiotics, an intraperitoneal injection of clindamycin (32 mg per kg) was given 1 day before *C. difficile* challenge. Then, 10_5_ CFU (in 100 μL of Chopped meat broth, a pre-reduced medium) of the vegetative *C. difficile* strain VPI10463 (ATCC 43255, tcdA+tcdB+cdtB-) was given by oral gavage (**Figure 1A**). The inoculum was prepared as previously described (30). Control mice received chopped meat broth (100 μL). Mouse weights and the development of disease symptoms were monitored daily. The animals were euthanized three days after infection using ketamine and xylazine (180 and 15 mg/kg, i.p.).

### Immunohistochemistry

Sections (4 µm thick) were prepared from paraffin-embedded mouse colon and cecum tissues. After deparaffinization, antigens were recovered by incubating the slides in citrate buffer (pH 9.0) for 20 min in PT link tank (DAKO). Endogenous peroxidase was blocked with 3% H2O2 for 30 min to reduce nonspecific binding. The sections were then incubated with an Panx-1 antibody (R&D system) for 3 hours. The sections were then incubated for 30 min with polymer (K4061, Dako). The antibody binding sites were visualized by incubating the samples with diaminobenzidine–H2O2 (DAB, Dako) solution. Sections incubated with antibody diluent without a primary antibody were used as negative controls. Immunohistochemical images were captured using a light microscope coupled to a camera with a LAZ 3.5 acquisition system (LEICA DM1000, Germany). To quantify the positive immunostaining area for Panx-1, the Adobe Photoshop 8.0 program was used to obtain the total tissue and the positive area. The percentage of immunopositive area was calculated by dividing the number of DAB-positive pixels (immunostaining-positive pixels) by the number of pixels per total tissue image and multiplying the result by 100, as previously described (36).

### Rat enteric glial cell culture and treatment

The immortalized rat enteric glial cell (EGC) line PK060399egfr (ATCC CRL-2690, VA, United States), which has been shown to exhibit similar morphology and functional properties to primary enteric glial cells (37), was cultured in Dulbecco’s Modified Eagle’s Medium (DMEM, Gibco) and supplemented with 10% fetal bovine serum, 1% antibiotics (100 μg/mL penicillin and 100 μg/mL streptomycin, Gibco) and 1 mM sodium pyruvate (Gibco) at 37°C in a humidified incubator under 5% CO2 for no more than 16 passages. For all experiments, EGCs were released using 0.05% trypsin-EDTA for 5 min. Cells were incubated with 10 and 50 µM 10Panx trifluoroacetate (Panx1 inhibitor, Sigma-Aldrich SML2152) or 300 µM A438079 (P2X7R antagonist, Tocris 2972) 1 h before incubation with TcdA (50 ng/mL) or TcdB (1 ng/mL). All drug concentrations used were based on MTT assay results (**Figure S1**). Purified TcdA and TcdB (TechLab, VA, United States) produced by *C. difficile* strain VPI10463 were used in this study.

### MTT assay

EGCs line (5x10^3^ cells/well) were seeded in 96-well plates and treated with 10Panx trifluoroacetate (10, 100 and 300µM) and A438079 (1, 3, 10, 30, 100 and 300μM) for 18h. Then, the cells were incubated with thiazolyl blue tetrazolium bromide (MTT, 0.5 mg/mL reconstituted in supplemented DMEM, Sigma-Aldrich, M2128) for 2 h at 37°C in a humidified incubator under 5% CO_2_. After removal of the MTT solution, 150 µL of dimethylsulfoxide was added to each well. The plates were then shaken for 2 min at room temperature, and the absorbance of the reaction at 570 nm was measured using an ELISA reader.

### Quantitative real-time PCR

EGCs line (6x10^5^ cells/well) were seeded in 6-well plates and treated with TcdA or TcdB and pharmacologic modulators. After incubation, total RNA was extracted with a RNeasy Plus Mini Kit (Qiagen, Hilden, Germany) using QIAcube (Qiagen). RNA was quantified with a Qubit 3.0 fluorometer (Life Technologies) using a Qubit RNA BR Assay Kit (Invitrogen, Q10211). After DNA contamination was removed by RNA treatment with DNase I (Invitrogen, 18068-015), a total of 600 ng of RNA was then reverse transcribed using an iScript cDNA Synthesis Kit (Bio-Rad, 1708891) according to the manufacturer’s protocol. qPCR amplification of Panx-1, IL-6 and glyceraldehyde 3-phosphate dehydrogenase (GAPDH) in cell samples was performed in a CFX Connect system (Bio-Rad) with the following conditions: 95°C for 30 s, 40 cycles of 95°C for 5 s and 60°C for 30s, and melt curve analysis from 65-95°C in 0.5°C increments for 2 s each. All PCRs were performed with iTaq Universal SYBR Green Supermix (Bio-Rad, 172-5124). The primer sets are listed in **Supplementary Material**.

### Immunofluorescence

EGCs line (4x10^4^ cells/well) plated on 8-chamber glass tissue culture slides in a polystyrene vessel and treated with TcdA or TcdB for 18 h were fixed in 4% PFA solution (Alfa Aesar) in PBS for 30 min at room temperature and permeabilized with 0.5% Triton X-100 (Sigma-Aldrich) and 3% bovine serum albumin (BSA, Sigma) in PBS for 10 min at 4°C. After blocking with 5% normal bovine serum albumin in PBS for 40 min at room temperature, the cells were incubated with anti-Panx-1 antibody (1:100, Invitrogen, 488100), anti-P2X7 antibody (1:50, Millipore, AB5246), anti-IL-6 (1:20, R&D system, AF506) or phosphorylated NFκB (1:100, Thermo Scientific, PA537718) overnight at 4°C. After three washes with washing buffer (0.01% Tween 20 in PBS), the cells were incubated for 2h with secondary antibody conjugated with Alexa Fluor 488 or 594 (1:400, Invitrogen, A21206/abcam, ab150129/abcam, ab150064) at room temperature, washed with PBS and mounted with ProLong Gold antifade reagent containing DAPI (Thermo Scientific, P36931). The samples were visualized by fluorescence microscopy (Zeiss).

### Caspase 3/7 activity assay

Caspase 3/7 activity was measured by using a Caspase-Glo assay kit (Promega, G8091) following the manufacturer’s instructions. EGCs (10^4^ cells/well) seeded in a white tissue culture-treated 96-well plates (Falcon, solid white bottom) were treated with TcdA or TcdB alone or in the presence with 10Panx (50µM) or A438079 (300µM) for 18 h. Then, the plates containing the cells were removed from the incubator for 30 min. A volume of 100 µL of Caspase-Glo reagent was added to each well, and the wells were mixed with a plate shaker at 500 rpm for 30 s. The plates were incubated for 2 h at room temperature. Then, the luminescence of each sample was acquired in a plate-reading luminometer (Promega) to obtain the relative luminescent units (RLUs) subtracted by the background.

### RealTime-Glo annexin V apoptosis assay

Apoptosis was evaluated with a live cell real-time assay (RealTime-Glo annexin V apoptosis assay, Promega, JA1000), following the manufacturer’s instructions. EGCs (10^4^ cells/well) were treated with TcdA or TcdB alone or in the presence of 10Panx (50 µM) or A438079 (300 µM) 1h prior to toxin challenge. Then, 200 µL of 2x detection reagent was added to each well, and the cells were incubated at 37°C in a humidified incubator under 5% CO_2_. The luminescence was recorded using a luminometer (NanoLuc technology ready, Promega), and the intrinsic reagent luminescence (no-cell, no-compound background control) was subtracted from the luminescence signals in the sample wells to obtain the relative luminescent units (RLUs).

### Measurement of extracellular ATP

Level of extracellular ATP was measured with a live cell real-time assay (RealTime-Glo extracellular ATP assay, Promega, GA5010), following the manufacturer’s instructions. EGCs (10^4^ cells/well) were treated with TcdA or TcdB alone or in the presence of 10Panx (50 µM) or A438079 (300 µM) 1h prior to toxin challenge. Then, 50 µL of 4x RealTime-Glo extracellular ATP assay reagent was added to each well, and the cells were incubated at 37°C in a humidified incubator under 5% CO_2_. The luminescence was recorded using a luminometer (NanoLuc technology ready, Promega), and the intrinsic reagent luminescence (no-cell, no-compound background control) was subtracted from the luminescence signals in the sample wells to obtain the relative luminescent units (RLUs) and the data was normalized by the control cells to obtain the fold change.

### Western blot analysis

EGC lines (6×10^5^ cells/well) were seeded in six-well plates and treated with TcdA or TcdB in the presence or absence of P2X7R antagonist (A438079) 1h prior to toxin challenge. After incubation, the supernatant was removed and the cells were lysed using RIPA lysis buffer (Thermo Fisher Scientific, containing EDTA and phosphatase-free protease inhibitor), centrifuged (17 min, 4°C, 13000 rpm) and the supernatant was collected. Protein concentrations were determined through the bicinchoninic acid assay according to the manufacturer’s protocol (Thermo Fisher Scientific). 40 µg of protein (previously prepared with Laemmil sample buffer and β-mercaptoethanol) were denatured at 95°C for 5 min, separated on 10% BIS-Tris gel and transferred to PVDF membranes for 2 h. After blocking with 5% blocking solution (BioRad) at room temperature for 1 h, the membranes were incubated overnight with the primary antibodies (anti-β-actin 1:500, Millipore EP1123Y and cleaved caspase-3 1:1000, Sigma-Aldrich PC679) at 4°C and secondary antibodies (anti-mouse 1:500 and anti-rabbit 1:500) for 1 h and 30 min. Membranes were washed in Tris-buffered saline containing 0.05% Tween 20 (TSB-T) and incubated with Enhanced Chemiluminescence – ECL (Biorad 1705060). The chemiluminescence signal was detected using a ChemiDoc system (BioRad). Densitometric quantification of bands was performed using ImageLab software (BioRad).

### Statistical analysis

Analyses were performed using GraphPad software 9.0 (San Diego, CA, USA). The data are presented as the mean ± standard error of the mean (SEM). Student’s t test or one- or two-way analysis of variance (ANOVA) followed by the Tukey test was used to compare means. *P*< 0.05 was considered to indicate significance.

## Results

### *C. difficile* toxins increase Panx1 expression in mouse intestinal tissues

Cecum and colon are the intestinal segments more affected by CDI in mice and in human (38–40). To investigate if levels of Panx1 in the intestine was affected by CDI, we performed an immunohistochemistry analysis. Using a CDI pre-clinical model (**Figure 1A**), we found increased positive immunostaining for Panx1 in the cecum (p = 0.01, **Figure 1B**) and in the colon (p = 0.04, **Figure 1B**) of infected mice compared to the control group (**Figure 1B**). Cecum and colon samples from mice with CDI showed notable increased immunostaining for Panx1 in the lamina propria and submucosa layer compared to the control group, together with epithelial cell disruption, inflammatory cell infiltrate, and submucosal edema (**Figure 1C**).

### *C. difficile* toxins increases Panx1 expression in EGCs *in vitro*

**Figure 2:**
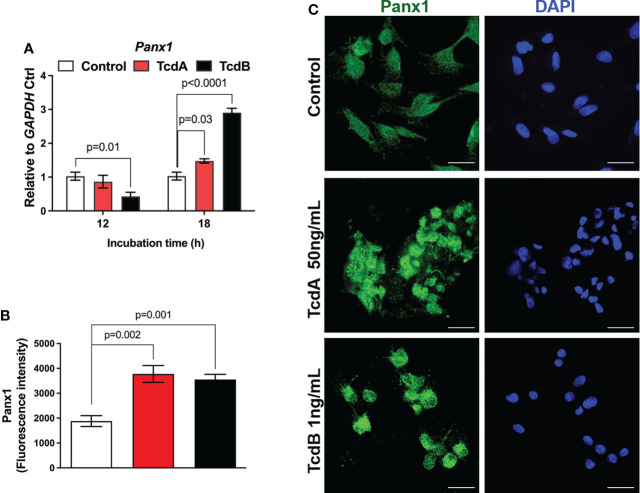
TcdA and TcdB alter Panx1 gene expression in EGCs *in vitro*. **(A)** Gene expression of *Panx1* were evaluated by qPCR in EGC incubated with DMEM only (control), TcdA (50 ng/mL) and TcdB (1 ng/mL). The data are the mean ± SEM. p value is represented in the graph; the one-way ANOVA test followed by the Tukey test was used. **(B)** Fluorescence intensity of Panx1 immunostaining in EGCs measured by ImageJ software. The data are the mean ± SEM. One-way ANOVA followed by the Tukey test was used. p value is represented in the graph. **(C)** Representative photomicrographs of Panx1 (green) immunostaining and DAPI (blue) nuclear staining in EGCs exposed to TcdA and TcdB after 18 h of incubation.

Next, we evaluated whether *C. difficile* toxins induce alteration on the gene expression of Panx1 in EGCs. We found that TcdA and TcdB upregulated the gene expression of *Panx1* in EGCs at 18h incubation compared to the control cells (p = 0.03 TcdA; p<0.0001 TcdB, **Figure 2A**). However, TcdB, but not TcdA, reduced the gene expression of *Panx1* at 12h incubation compared to the control group (p = 0.01, **Figure 2A**). The immunofluorescence data also showed increased positive immunostaining for Panx1 in EGCs incubated with TcdA and TcdB at 18h (**Figures 2B, C**). Due to the higher deleterious effects of *C. difficile* toxins occurred on the later time point, we focused on 18h incubation time, as shown by others (13, 14, 30).

### Panx1 inhibitor decreases EGCs death, but not *IL-6* expression, induced by TcdA and TcdB

**Figure 3:**
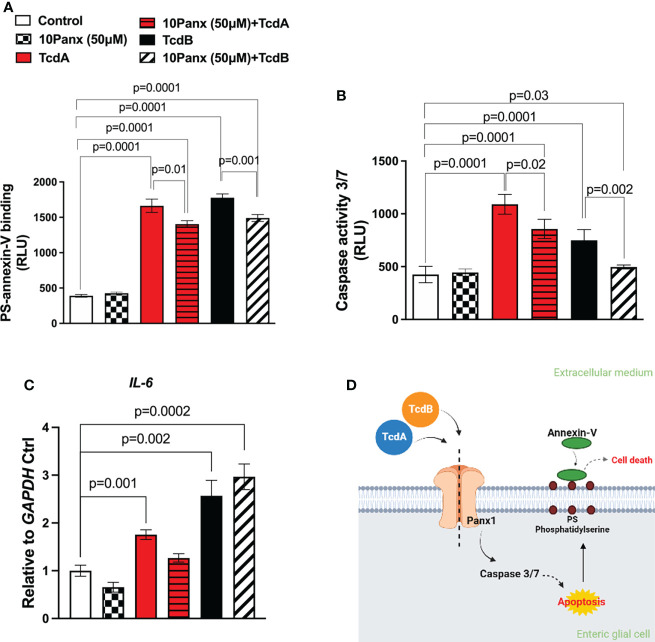
Panx1 inhibitor on caspase 3/7 activity and annexin V phosphatidylserine binding in EGCs challenged by TcdA and TcdB *in vitro*. **(A)** Cell death was analyzed by RealTime-Glo annexin V apoptosis assay in EGCs incubated for 18 h with TcdA, TcdB and 10Panx *trifluoroacetate* (10Panx; 50µM), a Panx1 antagonist, one hour prior to toxin challenge. For statistical analysis, the one-way ANOVA test was used followed by the Tukey test; the p value is represented in the graph. **(B)** The activity of caspase 3/7 was analyzed by luminescence assay in EGCs incubated for 18 h with TcdA, TcdB and 10Panx (50µM), one hour prior to toxin challenge. **(C)** *IL-6* gene expression was evaluated by qPCR in EGCs challenged by TcdA and TcdB for 18h, previously incubated or not with Panx1 inhibitor (10Panx, 50 μM). The data are the mean ± SEM. For statistical analysis, the one-way ANOVA test was used followed by the Tukey test; the p value is represented in the graph. **(D)** Proposed model of the role of Panx1 signaling in TcdA and TcdB-induced cell death in EGCs. TcdA and TcdB activate Panx1 in EGCs. Its activation results in the activation of caspase 3/7 and phosphatidylserine expose related to cell death.

To assess whether Panx1 participate on cell death and *IL-6* expression induced by *C. difficile* toxins in EGCs, we used a pharmacological approach (10Panx) to inhibit Panx1 before challenge EGCs with TcdA or TcdB. Inhibition of Panx1 (50 µM 10Panx) decreased phosphatidylserine-annexin V binding (p = 0.01 TcdA; p = 0.001 TcdB, **Figure 3A**) and the levels of caspase 3/7 activity (p=0.02 TcdA; p = 0.002 TcdB, **Figure 3B**), markers of cell death, induced by TcdA and TcdB in EGCs. The Panx1 inhibitor (50 µM 10Panx) did not prevent TcdA and TcdB-induced *IL-6* upregulation in EGCs (**Figure 3C**).

Taken together, these data demonstrated that Panx1 is involved in TcdA and TcdB-induced EGC death but not *IL-6* expression (**Figure 3D**).

### *C. difficile* toxins upregulates P2X7R expression in EGCs *in vitro*

**Figure 4:**
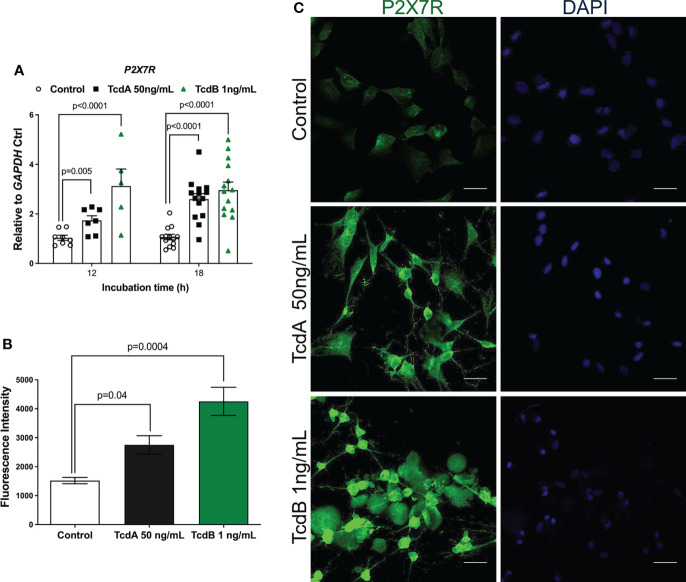
TcdA and TcdB upregulate P2X7R expression in EGCs. **(A)** Gene expression of *P2X7R* were evaluated by qPCR in EGCs incubated with DMEM only (control), TcdA (50 ng/mL) and TcdB (1 ng/mL). The data are the mean ± SEM. Two-way ANOVA followed by the Tukey test was used. **(B)** Fluorescence intensity of *P2X7*R immunostaining in EGCs measured by ImageJ software. The data are the mean ± SEM. One-way ANOVA followed by the Tukey test was used. p value is represented in the graph **(C)** Representative photomicrographs of P2X7R (green) immunostaining and DAPI (blue) nuclear staining in EGCs exposed to TcdA and TcdB after 18 h of incubation.

Once activated, Panx1 releases ATP, which in turn promotes P2X7R activation (41, 42). As shown in our **Supplementary Data**, inhibition of Panx1, in fact, decreased the levels of extracellular ATP in EGCs challenged by *C. difficile* toxins (**Figure S2A**). Next, we investigated if *C. difficile* toxins affected the expression of P2X7R. Our qPCR data showed that TcdA (p = 0.005) and TcdB (p<0.0001) upregulated *P2X7R* at 12h, which persisted at 18h incubation (p<0.0001) compared to control cells (**Figure 4A**). Confirming these findings, increased fluorescence intensity for P2X7R was found in EGCs challenged with TcdA and TcdB (p = 0.04 TcdA; p = 0.0004 TcdB, **Figures 4B, C**).

### P2X7R blockage decreases EGCs death induced by *C. difficile* toxins

**Figure 5:**
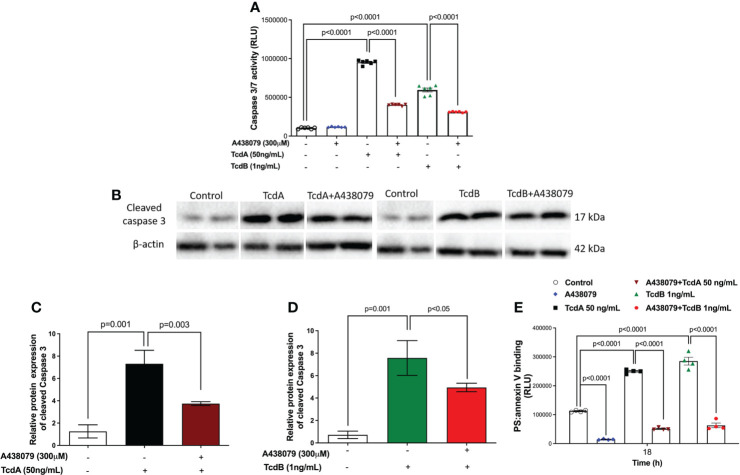
A438079, a P2X7R antagonist, prevents TcdA- and TcdB-induced EGCs death. **(A)** Activity of caspase 3/7 analyzed by luminescence assay in EGCs incubated for 18 h with TcdA, TcdB by qPCR. A438079, a P2X7R antagonist, was added to EGCs one hour prior to toxin challenge. **(B)** Representative Western Blot images showing the prevention of TcdA and TcdB induced cleavage of caspase 3 protein expression by A438079, a P2X7R antagonist. β-actin was used as a loading control. The WB images for the control group are the same for both toxins. **(C, D)** Graphs represents cleaved relative protein expression of caspase 3. The data are the mean ± SEM. One-way ANOVA followed by the Tukey test was used. **(E)** A438079 (P2X7R antagonist) prevented cell death analyzed by RealTime-Glo annexin V apoptosis assay in EGCs. The data are the mean ± SEM. Two-way ANOVA followed by Tukey test was used. p value is represented in the graph.

To determine whether P2X7R participated on the deleterious effects induced by TcdA and TcdB in EGCs, we inhibited this receptor using a selective antagonist (300 µM A438079) before challenging EGCs with the toxins. In fact, A438079 did not decrease the levels of extracellular ATP in EGCs challenged by *C. difficile* toxins (**Figure S2B**). Blockage of P2X7R markedly decreased the caspase 3/7 activity (p<0.0001, **Figure 5A**), as well as the levels of protein expression of cleaved caspase-3 (**Figures 5B–D**), in EGCs challenged by TcdA (p = 0.003) and TcdB (p<0.05). A438079 (P2X7R antagonist) also prevented cell death analyzed by phosphatidylserine-annexin V binding (p<0.0001, **Figure 5E**).

### Inhibition of P2X7R attenuates *IL-6* upregulation induced by *C. difficile* toxins in EGCs

**Figure 6:**
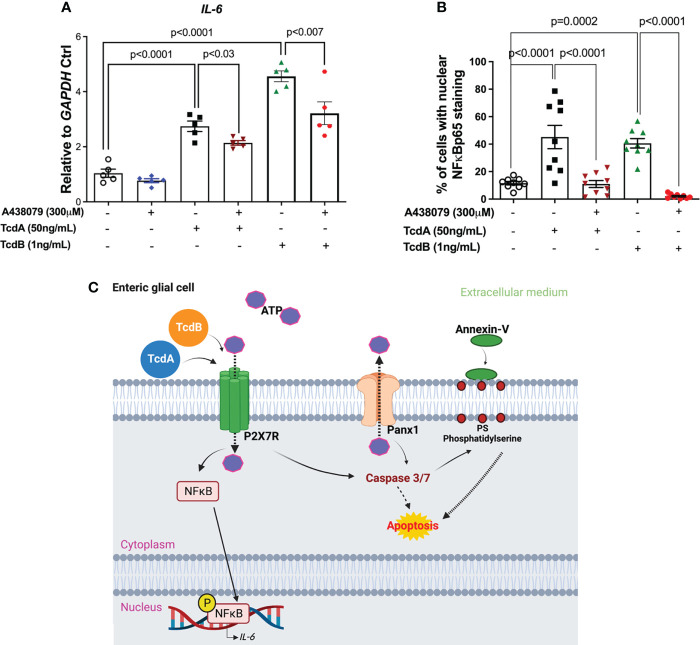
A438079, a P2X7R antagonist, inhibits TcdA- and TcdB-induced *IL-6* upregulation in EGCs. **(A)** *IL-6* mRNA expression in EGCs incubated for 18h with TcdA, TcdB and A438079 (300µM) 1h prior to toxin challenge, as analyzed by qPCR. The data are the mean ± SEM.; one-way ANOVA followed by the Tukey test was used. **(B)** Percentage of cells with positive nuclear phosphorylated NF*κ*Bp65 staining. The data are the mean ± SEM. p value is represented in the graph. **(C)** Proposed model of the role of Panx1/P2X7R signaling in TcdA- and TcdB-induced *IL-6* expression and in EGC death. TcdA and TcdB activate Panx1 in EGCs, promoting the release of ATP, which in turn activates P2X7R. Its activation results on nuclear translocation of NF*κ*B and *IL-6* expression, as well as caspase 3/7 activation that promotes cell death.

When we evaluated whether P2X7R participates on upregulation of *IL-6* induced by TcdA and TcdB on EGCs, we found that the inhibition of this receptor decreased *IL-6* gene expression induced by both toxins in the enteric glia (p<0.03 TcdA, p<0.007 TcdB, **Figure 6A**). Our protein analysis of IL-6 also showed similar findings (p<0.0001 TcdA, p=0.0003 TcdB, **Figures S3, S4**).

Finally, when we analyzed whether P2X7R blockage decreased the cells with nuclear phosphorylated NF*κ*B, we found that A438079 reduced the percentage of EGCs with positive nuclear NF*κ*Bp65 staining induced by TcdA and TcdB (p<0.0001, **Figures 6B**, **S4**).

Thus, these data together indicated that P2X7R is involved in EGCs death, as well as on upregulation of *IL-6* induced by both *C. difficile* toxins.

## Discussion

In the present study, high expression of Panx1 was found in colon and cecum samples from animals infected with *C. difficile*. The cecum, in fact, is the region of the intestine most affected by CDI (30, 38–40), with extensive rupture of the intestinal epithelium, hemorrhage, edema and recruitment of inflammatory cells. Increased Panx1 has been reported in several conditions, such as cerebral ischemia (43, 44), in metastatic cell lines of patients with breast cancer (45), in Crohn’s disease and in ulcerative colitis (9, 46).

We detected an increase in Panx1 protein levels on intestinal mucosa and submucosa layer. Given that EGCs is one of the ENS component that can be found on these two layers (6), we hypothesized that EGCs expressing Panx1 could have a role in CDI pathogenesis. Enteric glia is an intriguing population of cells that act modulating intestinal physiological processes such as motility, secretion, and maintenance of barrier function (9, 47). The importance of EGC on CDI has been demonstrated previously. Von Boyen et al. (48) showed that Glial fibrillary acidic protein (GFAP), an enteric glial marker is increased in colonic tissue from patients with CDI. Study from our group reported that S100B expression is increased in colonic biopsies and on fecal samples from patients with CDI, and in colon tissues from C. difficile-infected mice (30). We also demonstrated that S100B signaling is involved in EGC inflammatory response and that its inhibition attenuates the intestinal injury and diarrhea caused by C. difficile toxins (30).

The Panx1 channel establishes a communication between the cytosol and extracellular environment (17, 49). Furthermore, it is essential for the release of signaling molecules such as ATP (50–52). By detecting cellular danger signals, such as ATP, EGCs can be activated releasing other mediators that induce neuronal death and inflammatory response (47).

Here, the activation of Panx1 led to EGCs death induced by TcdA and TcdB. In an experimental colitis model, Panx1 was associated with enteric neurons death *via* caspase activation (9, 46) as also shown here. Cleaved caspase 3/7 target multiple cytoskeletal proteins, including key components of actin filaments, intermediate filaments, and microtubules (53). Both Caspase 3 and 7 are considered executor caspases, which are activated by initiator caspases arising from intrinsic or extrinsic apoptosis (54). It has been demonstrated that TcdA induces apoptosis in human epithelial cell line (T84) through intrinsic and extrinsic pathway *via* activation of initiator caspases 8 and 9 followed by activation of executor caspases 3 and 6, and bid cleavage (55). TcdB-promoted cell death has also been shown to occur *via* caspase 3 activation (13, 15, 56). The catalytic activity of executor caspases promotes cell death as they are responsible for a variety of morphological and biochemical changes that favor this process, such as DNA fragmentation, phosphatidylserine exposure and formation of apoptotic bodies (54, 57–59). In the present work, we demonstrated that Panx1 is involved in caspase 3/7 activation and in phosphatidylserine (PS) exposure. Caspase-3-mediated phosphatidylserine (PS) exposure through the activation of proteins related to its externalization, such as scrambling phospholipids, or inactivation of factors that promote its internalization, such as phospholipid flipases (60–62). Unlike caspase 3, which is the main caspase activated in the apoptosis process, caspase 7 is responsible for disintegrating cells from their extracellular component and their adhesion to other cells, as demonstrated in an *in vitro* study using embryogenic fibroblasts (63). Therefore, our data agree with previous demonstration that the opening of Panx1 channels can lead to cell death *via* release of ATP, which results in activation of purinergic receptors and increased intracellular calcium flow that stimulate the activation of initiator and effector caspases culminating in the apoptotic process (44, 49, 51, 54, 64).

Accordingly, we identified that both TcdA and TcdB stimulated the expression of P2X7R in EGCs. P2X7R has been extensively investigated due to its upregulation in a variety of diseases, such as Crohn’s disease, ulcerative colitis, systemic lupus erythematosus, depression and Alzheimer’s disease (65–69).

Interestingly, the P2X7R antagonist (A438079) decreased the EGCs death, as demonstrated here by reduced PS and annexin V binding and caspase 3/7 activity, showing the potential role of this receptor in promotes EGCs death induced by *C. difficile* toxins. The involvement of P2X7R in cell death has also been shown in other cells such as podocytes (70), macrophages (71), cerebellar astrocytes (72) and in neurons (73, 74). Mitogen-activated protein kinase (MAPKs) such as extracellular signal-regulated kinase (ERK) 1/2 and JNK have also been associated with cell death mediated by this receptor. In primary cortical neurons, P2X7R agonists mediate nuclear condensation and DNA fragmentation by expressing a functioning P2X7R and activating ERK1/2 and JNK1 in a manner dependent on caspase 8/9/3 activation (74). This could be one of the mechanisms of caspase 3 activation in EGCs challenged with TcdA and TcdB. In addition, a previous study showed that TcdB-induced EGCs apoptosis *via* NADPH oxidase/ROS/JNK/caspase-3 (15). Unlike this study that identified the intracellular signaling pathway involved in TcdB-induced EGCs death, here we showed a membrane receptor, P2X7R, involved in this process not only induced by TcdB, but also by TcdA.

However, further investigations are needed to verify the involvement of this receptor in CDI clinical outcomes, having in mind that drugs that block membrane receptors may be potentially more tolerable with fewer side effects than drugs that block components of the intracellular pathways, since they may participate in other processes important to the physiological functions of the cell.

Here we also show that Panx1 did not participate in *IL-6* upregulation induced by TcdA and TcdB. IL-6 can be synthesized by a variety of cells, including EGCs (30, 37, 75). IL-6 is a pleiotropic cytokine considered a predictor of CDI severity (76). The effects of IL-6 vary between promoting cell survival and pro-inflammatory effect (77–79). Consistent with our data, it was shown that Panx1 is not required for inflammasome activation, but participates in cell apoptosis (80, 81)

In contrast, P2X7R has been shown to regulate the expression and secretion of several cytokines and inflammatory mediators, including IL-2, IL-4, IL-13, IL-18, TNF-α, NO and IL-6 (82–86). Here, the P2X7R antagonist reduced the expression of *IL-6* induced by TcdA and TcdB. These data suggest the important role of this receptor in regulating the expression of this cytokine. In agreement with our findings, P2X7R has indeed been implicated in the regulation of IL-6 in various cell types including fibroblasts (87), skeletal muscle cells (88), macrophages (89) and microglia (90).

Our study is the first to show the involvement of Panx1/P2X7R signaling in cell death of EGCs challenged by TcdA and TcdB. TcdA and TcdB activate Panx1, releasing ATP, which in turn activates P2X7R leading to activation of caspase-3/7, culminating in exposure of PS in the plasma membrane, which characterizes EGC in process of cell death (**Figure 6C**). Further, activation of P2X7R promotes *IL-6* expression induced by *C. difficile* toxins. Further research on exploring Panx1/P2X7R signaling using pre-clinical model of CDI are needed to better understating the participation of these molecules in the pathogenesis of this disease.

## Data availability statement

The original contributions presented in the study are included in the article/**Supplementary Material**, further inquiries can be directed to the corresponding authors.

## Ethics statement

The animal study was reviewed and approved by Committee on the Ethics of Animal Experiments of the University of Virginia (Protocol number: 4096).

## Author contributions

AL, LM-N, DC, and GB wrote sections of the manuscript. AL and LM-N performed all the experiments and analyzed the data. CM, PS, ML, RL, JC-A, VM-N, and CW helped in the acquisition of data. AL, DC, and GB contributed to the organization of the manuscript, reviewed and approved the final version. All authors had full access, revised and approved the manuscript for publication.

## Funding

This study was funded by PRONEX CNPq/FUNCAP grant number PR2-0101-00060.01.00/15.

## Acknowledgments

The authors would like to thank Flávia A. Silva and Rosemayre Freire (analytics central/Federal University of Ceara) for their technical assistance.

## Conflict of interest

The authors declare that the research was conducted in the absence of any commercial or financial relationships that could be construed as a potential conflict of interest.

## Publisher’s note

All claims expressed in this article are solely those of the authors and do not necessarily represent those of their affiliated organizations, or those of the publisher, the editors and the reviewers. Any product that may be evaluated in this article, or claim that may be made by its manufacturer, is not guaranteed or endorsed by the publisher.
