# Supplementary material for: Role of Pannexin-1-P2X7R signaling on cell death and pro-inflammatory mediator expression induced by Clostridioides difficile toxins in enteric glia
Source: Front Immunol. 2022 Aug 22;13:956340. doi: 10.3389/fimmu.2022.956340 (PMC9442043; doi:10.3389/fimmu.2022.956340)
Supplement: Supplementary file 1 [file DataSheet_1.docx]

Figure S1


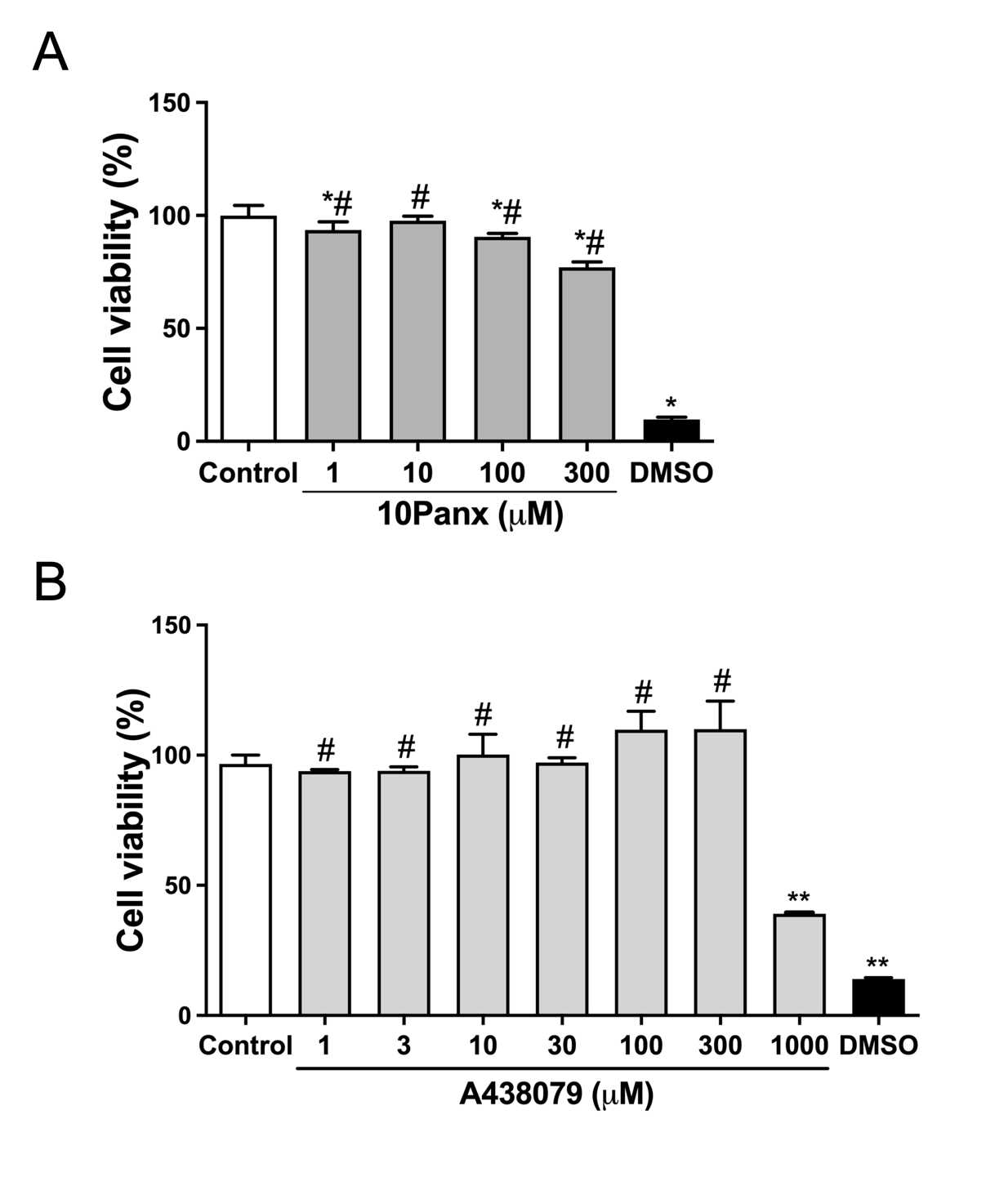


**Effects of 10Panx and A438079 on EGCs viability.** EGCs viability were assessed by MTT assay after 18h incubation with (A) 10Panx and (B) A438079. DMSO was used as a death control group. Data are presented as the mean ± s.e.m. (*n* = 6). *p<0.0001 versus control group and # p<0.0001 versus DMSO group. One-way ANOVA followed by Tukey test.

Figure S2


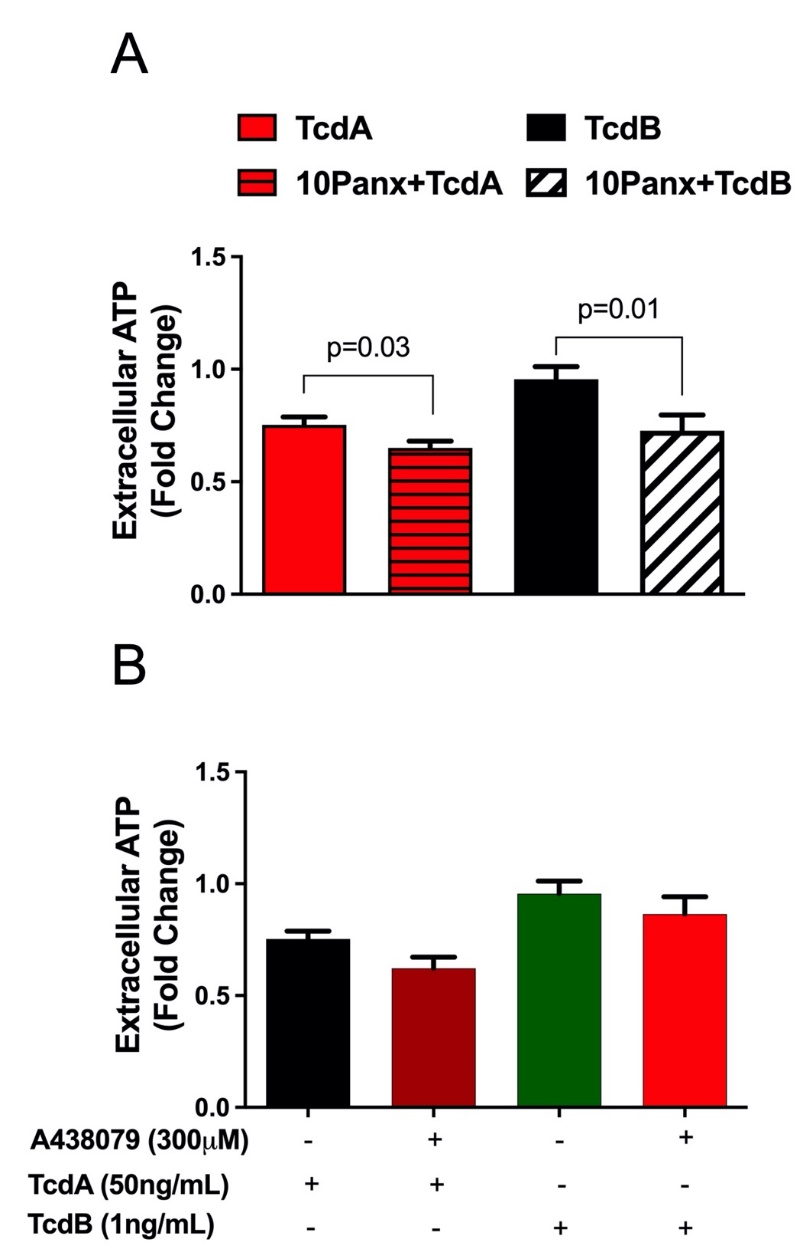


**Panx1 inhibitor, but not P2X7R antagonist, decreases levels of extracellular ATP in EGCs challenged by TcdA and TcdB in vitro.** (A-B) Levels of extracellular ATP were analyzed by RealTime-Glo assay in EGCs incubated for 18 h with TcdA, TcdB and (A) 10Panx *trifluoroacetate* (10Panx; 50µM), a Panx1 antagonist, or A438079, a P2X7R antagonist, one hour prior to toxin challenge. The data are the mean ± SEM. For statistical analysis, the one-way ANOVA test was used followed by the Tukey test; the p value is represented in the graph.

Figure S3


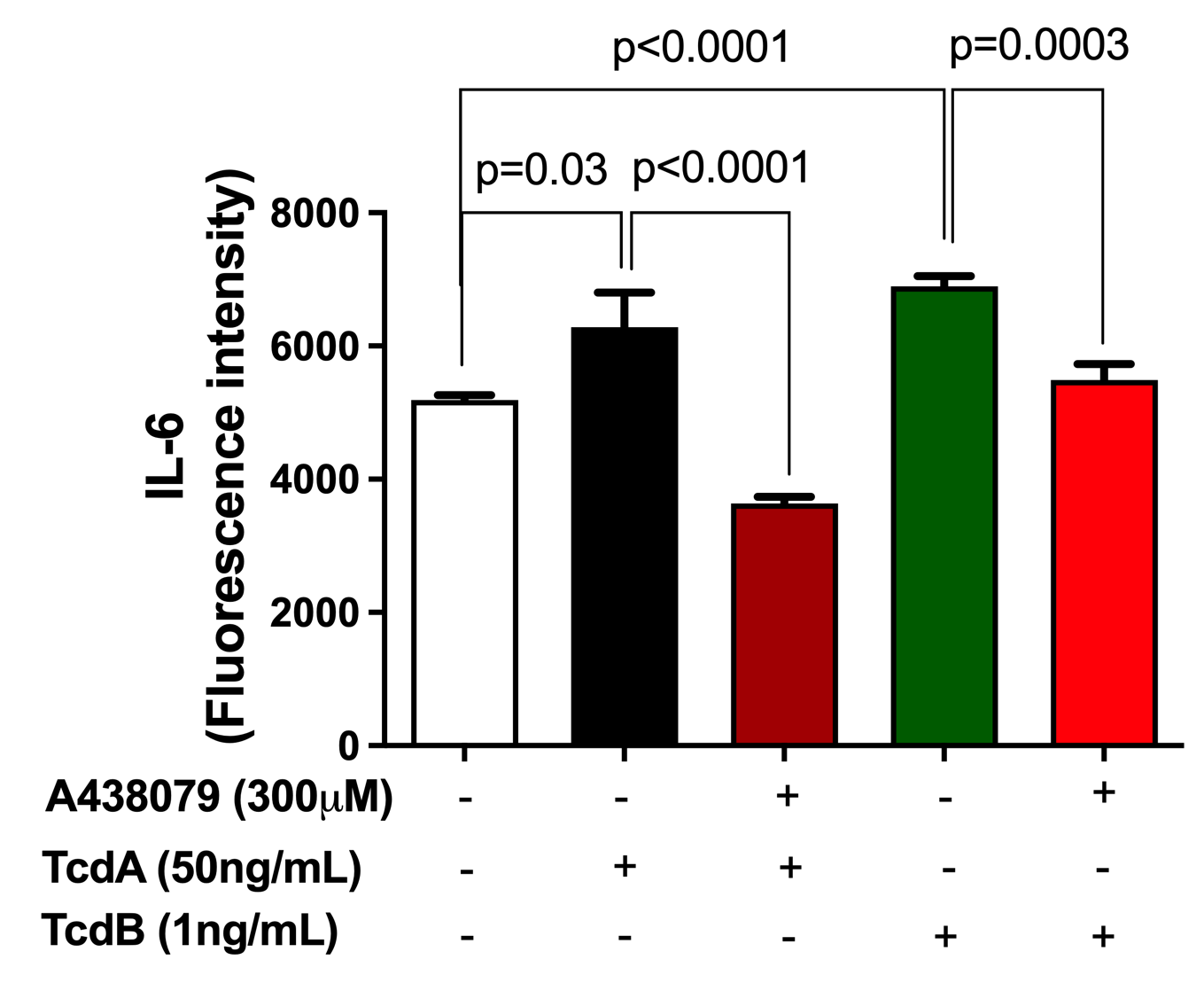


**A438079,** a **P2X7R antagonist, decreases TcdA- and TcdB-induced increase on IL-6 levels in EGCs.** Fluorescence intensity of IL-6 immunostaining measured by ImageJ software in EGCs incubated for 18h with TcdA, TcdB and A438079 (300µM) 1h prior to toxin challenge. The data are the mean ± SEM. One-way ANOVA followed by the Tukey test was used. p value is represented in the graph.

Figure S4


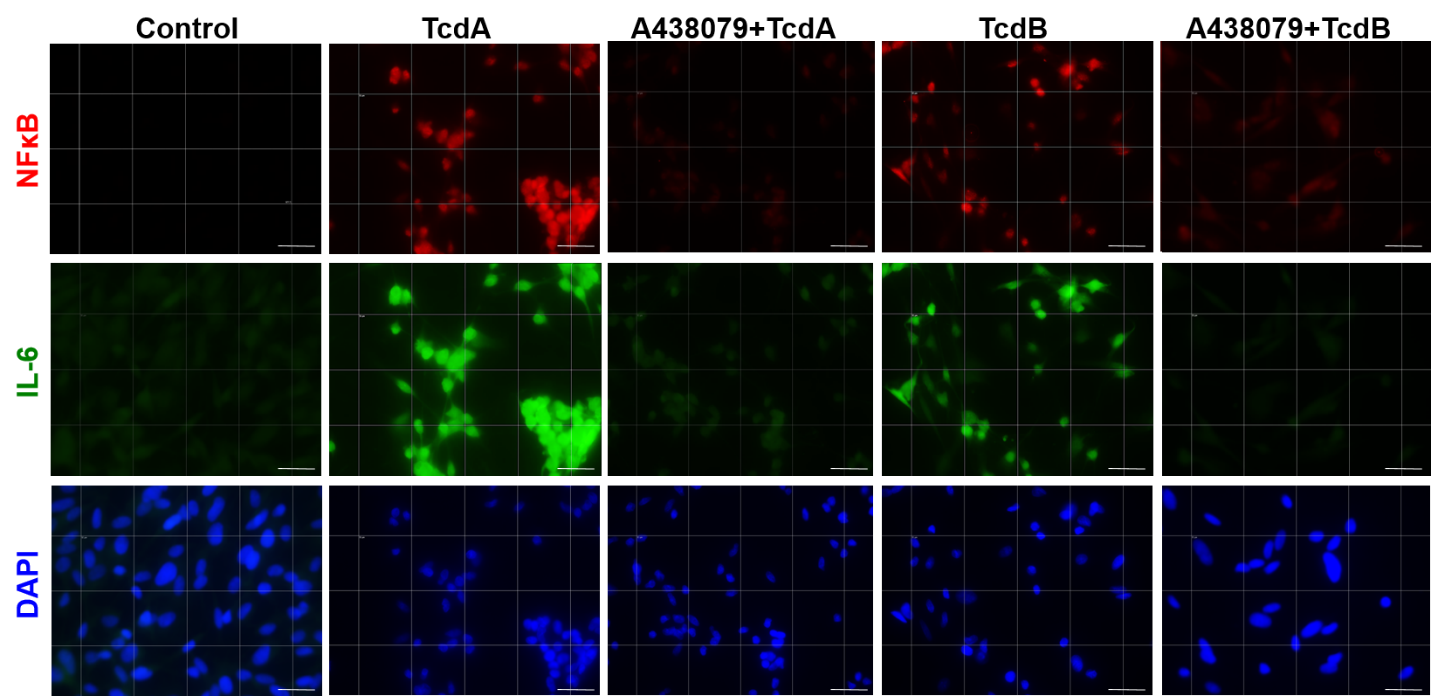


Representative photomicrographs of phosphorylated NF𝜅Bp65 (red) and IL-6 (green) immunostaining and DAPI (blue) nuclear staining in EGCs exposed to TcdA and TcdB after 18h of incubation and A438079, a P2X7R antagonist, one hour prior to toxin challenge.

Table 1 - Primers used in qPCR

| ***Panx1***  Forward CAAGGGAGAGGACCAGGGC  Reverse ATCTATTCTTCTATGACGCTG |
| --- |
| ***IL-6***  Forward GCCAGAGTCATTCAGAGCAATA  Reverse GTTGGATGGTCTTGGTCCTTAG |
| ***P2X7R***  Forward GAGCCCTGTGCAGTGAATGA  Reverse GCTCGTCCACAAAGGACACA |
| **GAPDH**  Forward AGACAGCCGCATCTTCTTGT  Reverse CTTGCCGTGGGTAGAGTCAT |
